# Special issue dedicated to Ørnulf Borgan

**DOI:** 10.1007/s10985-023-09592-w

**Published:** 2023-02-18

**Authors:** S. O. Samuelsen, O. O. Aalen

**Affiliations:** 1 Department of Mathematics, University of Oslo, Oslo, Norway; 2 Oslo Centre for Biostatistics and Epidemiology, Department of Biostatistics, University of Oslo, Oslo, Norway

Ørnulf Borgan celebrated his 70th birthday on April 8th, 2020, and retired from his Professorship at the University of Oslo that spring. A two-day symposium was originally planned in the spring of 2020 at the University of Oslo to celebrate Ørnulf’s many contributions within survival and event history analysis, counting process theory and case–control studies. The symposium had to be postponed because of the Covid-19 pandemic and was finally held on June 13th to 14th, 2022. The speakers at the symposium were invited to contribute to this special issue of Lifetime Data Analysis.

We are very happy to present this special issue of Lifetime Data Analysis with contributions from many of Ørnulf`s closest collaborators and colleagues. We are also grateful to Editor-in-Chief of LIDA, Mei-Ling Ting Lee, for allowing us to serve as Guest Editors for the issue.

Ørnulf Borgan has contributed to the theory and practice of life event history analysis for close to 50 years. He started out with a master's thesis at the University of Oslo in 1976 supervised by renowned insurance mathematician and demographer Jan M. Hoem. In 1977, Ørnulf followed Hoem to Copenhagen and became Assistant Professor at the Laboratory of Insurance Mathematics at the University of Copenhagen. While in Copenhagen he joined a group of researchers, formed around Niels Keiding, working with the fruitful framework of counting processes and martingale theory. Sadly, Niels passed away in the spring of 2022. The group, including their students, has been a driving force in the development of event history analysis ever since. It has also been a stronghold of statistics in Scandinavia.

After two and a half years in Copenhagen, Ørnulf returned to the University of Oslo and was Research Fellow in statistics until 1982. Then, for one year, he held an associate professorship at the Agricultural University of Norway (now the Norwegian University of Life Sciences) before he came back to the Department of Mathematics in 1984 first as Associate Professor and from 1992 as Professor. In 1984 Ørnulf received his Dr.Philos in statistics at the University of Oslo. Ørnulf was the Editor-in-Chief of the Scandinavian Journal of Statistics, 2007–2009 (together with Bo Lindqvist). He is Elected Member of the Royal Norwegian Society of Sciences and Letters, since 2019 and Elected Member of the Norwegian Academy of Science and Letters, since 2011, Fellow of the American Statistical Association, since 2015, and Elected Member of the International Statistical Institute, since 1997.

Ørnulf contributed to development of non-parametric tests based on counting processes (Andersen et al. 1982) and development of maximum likelihood properties for parametric models with right censored data using martingale theory (Borgan 1984). With Per Kragh Andersen he wrote a much cited paper summarizing the current state of the theory as of the mid-1980s (Andersen and Borgan 1985). Later he was one of the Andersen, Borgan, Gill and Keiding team presenting the extremely influential monograph Survival Models Based on Counting Processes (Andersen et al. 1993). He was also a coauthor on the text book Survival and Event History Analysis (Aalen et al. 2008).

From the 1990s Ørnulf's interest turned to analysis of outcome dependent sampling within survival data, in particular, extensions of nested case–control designs, and along with coauthors Bryan Langholz and Larry Goldstein he demonstrated the potential of counting process and martingale methods in this setting; see e.g. Borgan et al. (1995). This work, along with many other developments, was summarized in the Handbook of Statistical Methods for Case–control Studies edited by Borgan, Breslow, Chatterjee, Gail, Scott and Wild (Borgan et al. 2018).

Ørnulf has also been interested in methods for high-dimensional covariate data, so useful for gene expression covariate data, and has contributed to developments of survival analysis in this context, see e.g. Bøvelstad et al. (2009). Recently he has also been involved in development of machine learning methods for data with a time perspective (Kvamme et al. 2019).

Ørnulf Borgan is a thorough researcher, an excellent team worker and popular coauthor. You can always trust his contribution, be it a detailed technical work or data analysis. In addition, he is an excellent teacher and supervisor, establishing several popular courses, especially in survival analysis. Ørnulf is friendly, positive, interested, kind and modest.

Contained in this issue are papers on life history analysis reflecting many aspects of Ørnulf’s research interests. Several of these concern multistate models. Furberg, Andersen, Korn, Overgaard and Ravn write about recurrent event analysis with terminal events. They formulate a marginal model for recurrent events and terminal events simultaneously and show that the model can be analysed using bivariate pseudo-observations. Keogh, Diaz-Ordaz, Jewell, Semple, de Wrede and Putter discuss estimation of the distribution of length of stay, for different states in a multi-state model conditional on pathway. This work is motivated and applied to data on Covid hospitalization. Lindqvist extends phase-type modelling to competing risk settings assuming a finite state Markov chain with more than one absorbing state and considers identifiability of parameterizations in this context.

Goldstein and Langholz present a generalization of work with Ørnulf from 1995 on large sample theory for generalized nested case–control data where they allow for fine stratification of the cohort with different baseline hazards in each stratum. Hjort and Stoltenberg present work on additive hazards models where some of the regression functions are assumed to be parametrically specified, whereas others are left unspecified. One of us (Samuelsen) discusses collapsibility in proportional and additive hazards models demonstrating that even though the proportional hazards model is non-collapsible the Cox-estimator can still be collapsible. Conversely, even though the additive hazards model is collapsible, estimation in such models need not be. De Bin and Stikbakke present implementation of boosting for first hitting time models with high-dimensional covariates and apply such methods to gene expression data sets also analyzed by Bøvelstad, Nygård and Borgan (Bøvelstad et al. 2009). Blanche, Holt and Scheike discuss using logistic regression for right censored and competing risk data with two different inverse weighting schemes, handling the censoring, both for standard survival and competing risk situations.
